# A Visit to the Massachusetts General Hospital

**Published:** 1870-01

**Authors:** 


### Correspondence.

### A Visit to the Massachusetts General Hospital.

*Editor Buffalo Medical and Surgical Journal:*

Dear Sir:—Not many months since a long-cherished desire of mine was gratified in a visit to this institution, some little account of which I have thought would not be wholly devoid of interest to the readers of the Journal. Medical practitioners at home have a way of familiarizing themselves with hospitals abroad to a greater extent than with their own institutions at home. Physicians may have considerable knowledge of Guy's Hospital in London, through the medium of "Guy's Hospital Reports," while knowing but little of Bellevue, the Pennsylvania or the Massachusetts General Hospitals, which institutions have not until recently issued "Reports."

We now are in possession of the means of knowing more of the Pennsylvania Hospital, since the staff have put into our hands the first and second volumes of the *Pennsylvania Hospital Reports,* having begun this good work—with promise of continuance—we may hereafter know as much of the medical and surgical practice in at least one of our hospitals, as we do of that within the walls of hospitals abroad.

The Massachusetts General Hospital has attained to nearly sixty years of age, but is not in its dotage by any means. It has very recently provided itself with one of the finest operating rooms to be found in any hospital in any country. It was my pleasure to visit every part of the hospital from the surgical and medical wards down into the laundry and kitchen, to observe not only how space was economized in the plan of drying clothes, but to witness the process of soup making. The visitor I think, in first entering within the walls of this institution, would be forcibly impressed with its cleanliness and quaint appearance. The old fashioned fire places will attract his notice, his almost noiseless tread upon solid pine flooring in the stair way, halls and wards, must certainly be grateful to all inmates. The system of ventilation I conclude is perfect, not a disagreeable odor was detected during my presence within the hospital.

By the kindness of Dr. George H. Gay, one of the attending surgeons, I made the acquaintance of Drs. Cabot and Bigelow, both of whom are also visiting surgeons; as it was ten o'clock, and the service of Dr. Cabot, by invitation we accompanied this gentleman through the surgical wards into the operating rooms and witnessed some minor operations. I saw within the surgical wards more stumps from amputations, single and double, of both upper and lower extremities, than I have before witnessed in any other hospital. I had just previously visited Bellevue, New York, but I should judge that the relative number of amputations in the two institutions, at the time of my visit, was as ten in the former to one in the latter. This number, which seemed greatly disproportioned to the capacity of the two institutions, led me to inquire how it happened—why so many amputations, and if what I saw before me was what we sometimes used to style "conservative surgery," and was answered that the patients brought there were injured by the rail roads, and that the injuries were so severe from this cause as to necessitate amputation; and in this connection I was told what, however, I before knew, that the rail roads formed a perfect net-work in and around Boston; that the roads diverged in every direction from Boston, as a centre, like the spokes of a wheel, and in this way I conjecture Boston had come to enjoy the *sobriquet* of "Hub."

In this hospital, dressing for compound fractures consists of acid carbolic and glycerine, equal parts, with white lead enough to make a paste, over which is placed tin foil.

Dextrine is used for splints in place of the starch bandage. It dries quickly and makes the bandage as hard as board.

Ferri per sulph. is no longer used as an injection for the cure of varicose veins, on account of the injury it had in several cases inflicted upon the patients. The caustic potassa is employed instead.

Upon entering the female surgical wards, we were met by two female medical students, who were privileged to visit these wards with the visiting surgeons, and to receive such instruction at the bedside as the female patients could furnish and the visiting surgeons and physicians could impart.

At the end of a well spent two hours in this hospital, I was invited to be present at the opening lecture of the Medical Department of Harvard. As the institution is adjacent to the hospital, I availed myself of the kind invitation and was present at the opening lecture.

Upon the platform were assembled the savans of Cambridge and the *literati* of Boston to the number of thirty or more, among whom was Prof. Agassiz. The audience room proper was filled to overflowing; there was not even standing room, and many had to go away disappointed in not being able to hear the opening lecture.

I learned, there were an increased number of students in attendance, and they were certainly as intelligent a body of students as I ever saw assembled together.

I could not avoid the conviction that Boston was a most desirable place for young men to acquire a thorough knowledge of the medical profession, and shall ever remember my visit there as one among the most pleasant of my life, and shall gratefully cherish the kindness and courtesy shown me by those whom I was thrown in contact. Yours, etc., G.

*Buffalo.*

